# Supplementary material for: Nanoporous Iron Oxide/Carbon Composites through In-Situ Deposition of Prussian Blue Nanoparticles on Graphene Oxide Nanosheets and Subsequent Thermal Treatment for Supercapacitor Applications
Source: Nanomaterials (Basel). 2019 May 21;9(5):776. doi: 10.3390/nano9050776 (PMC6566787; doi:10.3390/nano9050776)
Supplement: Supplementary file 1 [file nanomaterials-09-00776-s001.pdf]

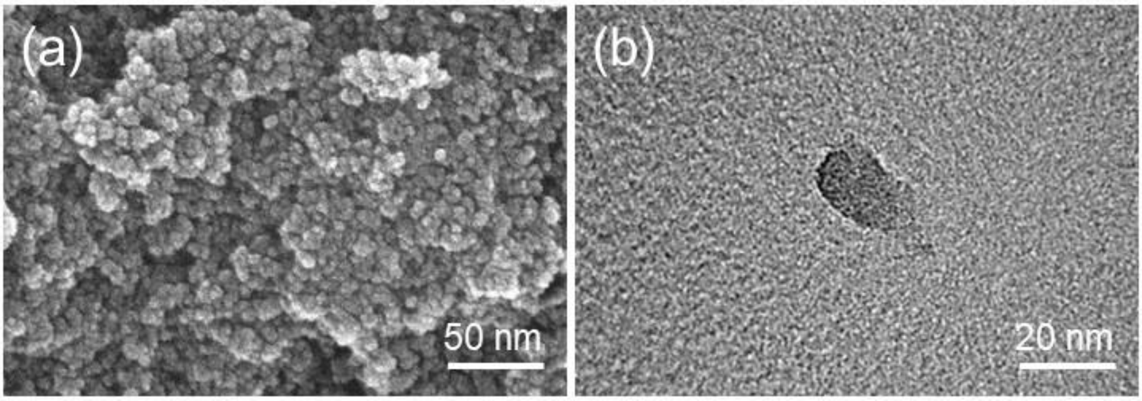


**Figure S1.** (**a**) SEM and (**b**) TME images of PB nanoparticles.

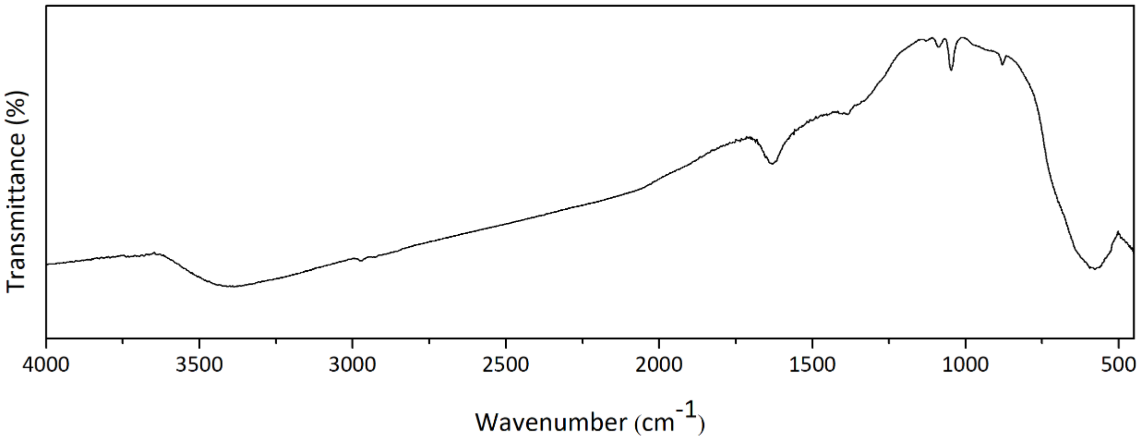


**Figure S2.** FTIR spectrum of iron oxide/carbon composite.

**Note in Figure S2:** The characteristic peaks of C=O and a broad peak of -OH disappeared, due to decreasing in various oxygen-containing functional groups [S1,S2]. The peak around 1600 cm$^{-1}$ can be referred to the C=C band. The C≡N bond also disappeared, indicating to remove the C-N group by calcination. The strong vibration bands at 577 cm$^{-1}$ was assigned to iron oxide ($Fe_2O_3$)[S3,S4], which indicates that the conversion of PB to iron oxide during the heat treatment.

**Table S1.** Comparison of our sample performance with the previously reported iron oxide/carbon materials composites for supercapacitors.

| Composition | Electrolyte | Capacitance ($F g^{-1}$) | Scan rate ($mV s^{-1}$) | Current density ($A g^{-1}$) | Reference |
|---|---|---|---|---|---|
| γ-$Fe_2O_3$/carbon | 3.0 M KOH | 551.5 | 2.0 | --- | This work |
| γ-$Fe_2O_3$/carbon | 3.0 M KOH | 415.0 | --- | 2.0 | This work |
| GO/iron oxide | 3.0 M KOH | 91.0 | 20.0 | --- | [S5] |
| N-rGO/$Fe_2O_3$ | 1.0 M KOH | 268.4 | --- | 2.0 | [S1] |
| $Fe_2O_3$-graphene | 2.0 M KOH | 151.8 | --- | 1.0 | [S1] |
| $Fe_2O_3$-rGO | 1.0 M $Na_2SO_4$ | 226.0 | --- | 1.0 | [S1] |
| GNS/$Fe_2O_3$ | 6.0 M KOH | 320.0 | --- | 10.0 | [S1] |
| g-$C_3N_4$/α-$Fe_2O_3$ | 2.5 M $Li_2SO_4$ | 260.0 | --- | 0.5 | [S1] |
| GS/$Fe_3O_4$ | 1.0 M KOH | 368.0 | --- | 1.0 | [S6] |
| GS/$Fe_3O_4$ | 1.0 M KOH | 245.0 | --- | 5.0 | [S6] |
| $Fe_3O_4$/graphite | 0.5 M $Na_2SO_4$ | 450.0 | 10.0 | --- | [S7] |
| $Fe_3O_4$/graphite | 0.5 M$Na_2SO_4$ | 327.3 | --- | 1.5 | [S7] |
| $Fe_2O_3$/GA | 0.5 M $Na_2SO_4$ | 81.3 | --- | 1.0 | [S8] |
| $Fe_3O_4$ sheets/rGO | 6.0 M KOH | 193.4 | --- | 0.3 | [S9] |


[S1] Liu, H. D.; Zhang, J. L.; Xu, D. D.; Huang, L. H.; Tan, S. Z.; Mai, W. J. *J. Solid State Electrochem.* **2014**, *19*, 135.

[S2] Zhu, Y.; Chu, W.; Wang, N.; Lin, T.; Yang, W.; Wen, J.; Zhao, X. S. *RSC Adv.* **2015**, *5*, 77958.

[S3] Zhu, K.; Chen, C.; Xu, M.; Chen, K.; Tan, X.; Wakeel, M.; Alharbi, N. S. *Chem. Eng. J.* **2018**, *331*, 395.

[S4] Jing, Z.; Wu, S. *J Solid State Electrochem.* **2004**, *177*, 1213.

[S5] Tanaka S.; Salunkhe R. R.; Kaneti Y. V.; Malgras, V.; Alshehri, S. M.; Ahamad, T.; Zakaria, M. B.; Dou, S. X.; Yamauchi, Y. *RSC Adv.* **2017**, *7*, 33994.

[S6] Liu, M.; Sun, J. *J. Mater. Chem. A* **2014**, *2*, 12068.

[S7] Sayahi, H.; Mohsenzadeh, F.; Darabi, H. R. *J. Alloys Compd.* **2019**, 778, 633.

[S8] Song, Z.; Liu, W.; Xiao, P.; Zhao, Z.; Liu, G.; Qiu, J. *Mater. Lett.* **2015**, 145, 44.

[S9] Yan, F.; Ding, J.; Liu, Y.; Wang, Z.; Z. Cai, Z.; Zhang, J. *Synth. Met.* **2015**, 209, 473.
